# Epigenetic therapy for ovarian cancer: promise and progress

**DOI:** 10.1186/s13148-018-0602-0

**Published:** 2019-01-15

**Authors:** Sara Moufarrij, Monica Dandapani, Elisa Arthofer, Stephanie Gomez, Aneil Srivastava, Micael Lopez-Acevedo, Alejandro Villagra, Katherine B. Chiappinelli

**Affiliations:** 1 Department of Microbiology, Immunology, & Tropical Medicine, The George Washington University, Washington, D.C., 20052 USA; 2 Department of Obstetrics & Gynecology, The George Washington University, Washington, D.C., 20052 USA; 3 Department of Biochemistry & Molecular Medicine, The George Washington University, Washington, D.C., 20052 USA; 4 The George Washington Cancer Center, The George Washington University, Washington, D.C., 20052 USA

**Keywords:** Ovarian cancer, Epigenetics, DNA methylation, Histone modifications, DNMT inhibitors, HDAC inhibitors

## Abstract

Ovarian cancer is the deadliest gynecologic malignancy, with a 5-year survival rate of approximately 47%, a number that has remained constant over the past two decades. Early diagnosis improves survival, but unfortunately only 15% of ovarian cancers are diagnosed at an early or localized stage. Most ovarian cancers are epithelial in origin and treatment prioritizes surgery and cytoreduction followed by cytotoxic platinum and taxane chemotherapy. While most tumors will initially respond to this treatment, recurrence is likely to occur within a median of 16 months for patients who present with advanced stage disease. New treatment options separate from traditional chemotherapy that take advantage of advances in understanding of the pathophysiology of ovarian cancer are needed to improve outcomes. Recent work has shown that mutations in genes encoding epigenetic regulators are mutated in ovarian cancer, driving tumorigenesis and resistance to treatment. Several of these epigenetic modifiers have emerged as promising drug targets for ovarian cancer therapy. In this article, we delineate epigenetic abnormalities in ovarian cancer, discuss key scientific advances using epigenetic therapies in preclinical ovarian cancer models, and review ongoing clinical trials utilizing epigenetic therapies in ovarian cancer.

**Electronic supplementary material:**

The online version of this article (10.1186/s13148-018-0602-0) contains supplementary material, which is available to authorized users.

## Background and main text

### Genetic aberrations in ovarian cancer

Ovarian cancer is a disease characterized by late-stage presentation and poor prognosis. Women often present with “silent symptoms” including abdominal bloating and pain, causing delayed referral for workup of a malignancy [1]. The risk factors of nulliparity, early-onset menarche, and late onset menopause and the protective factors of increased parity, increased time lactating, and use of oral contraceptive pills imply that ovarian cancer risk is proportional to the number of ovulations in a lifetime [2]. Besides ovulation number, family history is a strong risk factor. Hereditary predisposition is responsible for 14–24% of ovarian cancers, with the majority attributable to inherited mutations in the *BRCA1* or *BRCA2* genes [3]. BRCA1 and BRCA2 proteins are involved in repair of double strand DNA breaks by homologous recombination [4]. Loss of function of *BRCA1* or *BRCA2* or genes encoding proteins that complex with BRCA proteins, such as *BRIP1*, *RAD51C*, *RAD51D*, and *FANCM*, leads to genomic instability. Genomic instability is a hallmark of high-grade serous epithelial ovarian cancers, with early somatic mutations found in the tumor suppressor *TP53* [5]. Similarly, mutations in the mismatch repair (MMR) genes *MLH1*, *MSH2*, *MSH6,* and *PMS2* are associated with Lynch syndrome. Loss of MMR function leads to genomic instability, increasing the risk for gastrointestinal malignancies, endometrial cancer, and ovarian cancer, with increased representation in the endometrioid and clear cell types [6]. The genetic underpinnings of approximately half of ovarian cancers have not yet been characterized and are thought to be due to multiple alleles, potentially including genetic variants that are common in the general population [5]. Confirmed susceptibility loci that are common variants have been described in non-protein coding regions of the genome, suggesting a regulatory role of these regions [7].

### Histological subtypes of ovarian cancer and their molecular characteristics

Epithelial ovarian cancer (EOC) represents the largest subgroup (90%) of ovarian cancers. EOCs are distinguished by histology, of which papillary serous is the most common (75%) [2]. Serous carcinomas are further subdivided into high-grade and low-grade tumor types. High-grade and low-grade serous carcinomas behave differently in terms of disease progression and response to platinum-based chemotherapy: low-grade serous carcinomas (LGSC) are often associated with borderline serous tumors, implying that they may arise from precursor lesions. LGSCs tend to follow a more indolent course and are relatively platinum-resistant, compared to high-grade serous tumors which are often aggressive and can respond to platinum therapies [7].

High-grade serous carcinomas (HGSC) are the most common serous tumors. Over 90% of high-grade serous ovarian cancers harbor somatic *P53* mutations. The majority of *P53* mutations found in ovarian cancer are missense mutations, most of which occur in the DNA-binding domain of the protein. This is also the domain through which P53 exerts its major function as a tumor suppressor, by trans-activating target genes regulating cell cycle progression, proliferation, and apoptosis. *P53* mutations not only deplete wild-type P53 tumor-suppressive functions but can also act in a dominant-negative fashion on tetramerization of wild-type P53 with its target DNA sequence. In addition, the mutant P53 protein frequently acquires an oncogenic gain-of-function in these tumors leading to uncontrolled proliferation, increased metastatic potential, and higher risk of acquiring resistance to specific therapies, all through transcriptional regulation of genes important for tumorigenesis, cancer progression, and metastasis [8, 9].

Low-grade serous tumors, which include low-grade serous carcinoma and serous borderline tumors, are distinguished by their low mitotic rate and mild to moderate nuclear atypia in comparison to the high mitotic rate and marked nuclear atypia seen in HGSC [7]. In contrast to the high frequency of *P53* mutations in HGSC, *P53* mutations are significantly less frequent in LGSC and serous borderline tumors [10]. Furthermore, the Mitogen-Activated Protein Kinase (MAPK) pathway plays an important role in the pathogenesis of LGSC tumors. Singer et al. found mutations in *BRAF* and *KRAS*, kinases that activate oncogenic MAPK signaling*,* in approximately 60% of LGSC tumors, but none in HGSCs [11].

The oncogenes *KRAS* and *PI3K* as well as the tumor suppressor genes *PTEN* and *ARID1A* are mutated in other EOCs including clear cell, endometrioid, and mucinous tumors [12]. Mucinous ovarian cancers (mOC) are unique in their presentation and genetic composition. The predominant mutations found in mOC are *KRAS* mutations, which can also occur in benign ovarian tissue, borderline mucinous tumors, and malignant mucinous tumors, suggesting a benign-to-malignant progression driven by KRAS signaling [13]. mOC demonstrate platinum resistance and are not associated with *P53* mutations or inherited *BRCA* mutations [12]. A recent shift in the National Comprehensive Cancer Network (NCCN) guidelines has included chemotherapies typically used for treatment of colon cancer as an option for first line treatment of mOC, given the molecular similarities to colon cancer and the demonstrated benefits of this treatment method in mOC [14].

Mutations in the chromatin modulator *ARID1A* have been implicated in endometrioid ovarian cancers and clear cell carcinomas. *ARID1A* encodes the protein BAF250a, which is a component of the SWI-SNF chromatin remodeling complex. This complex is important for tumor suppression, DNA repair, and growth regulation, and is mutated in a wide variety of human cancers [15]. Ovarian cancers with *ARID1A* mutations are particularly sensitive to epigenetic modulators, as will be discussed in detail below. Our improved understanding of genetic aberrations in ovarian cancer is now allowing for the development of tailored treatment approaches, a relatively recent shift for this disease.

### Targeted therapies for ovarian cancer

For EOC, first line treatment is surgery for staging disease and cytoreduction to reduce tumor volume, followed by cytotoxic chemotherapy with a platinum agent and a taxane, which can be given in the neoadjuvant (before surgery) or adjuvant (after surgery) setting. EOC that recurs within 6 months of completion of initial therapy is considered “platinum-resistant,” while EOC that recurs after 6 months of completion of initial therapy is considered “platinum-sensitive” [14]. Targeted therapies use drugs that target pathways crucial for the growth or survival of ovarian cancer cells. These drugs target specific proteins, reducing the adverse effects on normal cells that frequently occur with cytotoxic chemotherapy [16]. Targeted therapies are most commonly first evaluated in the setting of recurrent disease, then may be studied further as a component of first-line treatment if success is demonstrated in clinical trials focused on recurrent disease [2].

P53 is the most commonly mutated protein in HGSC. Several compounds have been developed that aim to restore the functionality of P53 in cancers. The drug APR-246 binds to P53 via cysteine 277, leading to thermostabilization of wild-type and mutated P53 [17–20]. APR-246 is currently being evaluated in several early-phase clinical trials, two of which are on HGSC patients with P53 mutations in combination with full-dose chemotherapy (NCT02098343 and NCT03268382). Early results of those studies indicate that the drug was well tolerated and showed a favorable safety profile with only mild side effects. Nutlin-3a inhibits MDM-2, a negative regulator of P53, to increase P53 expression. Early clinical data demonstrated that an oral formulation of Nutlin-3a, RG7112, is well-tolerated in patients with initial evidence of clinical activity [21, 22]. Despite these early promising clinical trials, it remains to be shown whether drugs reactivating mutant P53 or increasing wild-type P53 are viable in the clinic and show superior efficacy for the treatment of human ovarian cancer over standard therapy.

Another targeted therapy in ovarian cancer includes molecules that inhibit angiogenesis. As tumors grow, they tend to become hypoxic, triggering the formation of new blood vessels, also known as angiogenesis. Signaling between vascular endothelial growth factor (VEGF) and its receptor (VEGF-R) promotes endothelial cell proliferation and migration [16]. Abu-Jawdeh et al. showed that angiogenesis plays a role in the initiation and progression of ovarian carcinogenesis [23]. Bevacizumab is a monoclonal antibody against VEGF-A, a member of the VEGF growth factor family, that was first approved by the Food and Drug Administration (FDA) in 2004 for the treatment of metastatic colon cancer. After several phase II studies demonstrated that bevacizumab could improve progression-free survival (PFS) in recurrent ovarian cancer, larger phase III trials investigated its application in the initial treatment setting as well as in the recurrent setting for both platinum-sensitive and platinum-resistant ovarian cancers. Two clinical trials, ICON7 (NCT00483782) and GOG218 (NCT00262847), showed that the addition of bevacizumab as initial therapy with continuation as maintenance therapy improved PFS. These effects were promising, but since they were modest and associated with an increased risk of bowel perforation (increased above the risk seen in colon cancer treated with bevacizumab), bevacizumab was not routinely adopted as first-line therapy.

The observation that EOC patients with germline *BRCA1* or *BRCA2* mutations had improved overall survival led to the idea of enhanced sensitivity to platinum agents due to loss of DNA repair [24]. BRCA1 or BRCA2 loss of function impacts the ability of the cell to repair double-stranded DNA breaks. Poly (ADP ribose) polymerase (PARP) is critical for the repair of single-stranded DNA damage; thus, PARP inhibitors prevent the cell from repairing single-stranded DNA breaks. Loss of either DNA repair function leads to genomic instability. PARP inhibitors cause loss of both pathways in BRCA-deficient tumors in an example of “synthetic lethality”. The SOLO2 trial demonstrated in 295 *BRCA* carriers with relapsed platinum-sensitive ovarian cancer that PFS was significantly longer with the PARP inhibitor olaparib (19.1 months [95% CI 16.3–25.7]) compared to placebo (5.5 months [95% CI 5.2–5.8]; *p* < 0.0001) [25]. The benefits of PARP inhibitors do not appear to be limited to *BRCA* carriers, as other trials have demonstrated promising results with the use of PARP inhibitors in patients with wildtype *BRCA* [26].

Finally, epidermal growth factor receptor (EGFR) is commonly overexpressed in ovarian cancer and associated with poor prognosis [2]. Cetuximab, a chimeric IgG1 monoclonal antibody that binds to the extracellular domain of EGFR, produced an objective response in 9 out of 26 patients when combined with carboplatin in patients with EGFR-positive ovarian or primary peritoneal cancer. However, the response rate did not support a second stage of patient selection [27].

### Immunotherapy for ovarian cancer

Ovarian cancer is characterized by a highly immunosuppressive microenvironment. The production of VEGF further aids tumor growth and angiogenesis. Tumors inhibit dendritic cells, specialized antigen-presenting cells, which typically prime immune cells to fight invading pathogens and/or cancer cells [28]. Tumor cells can secrete factors such as transforming growth factor beta (TGF-ß) that suppress cytotoxic CD8 T cells that can kill tumors. Tumor cells may also express immunosuppressive ligands, such as programmed cell death ligand-1 (PD-L1), which suppresses T cells by binding to its receptor programmed cell death protein 1 (PD-1) and results in immune tolerance. Immune checkpoint proteins like PD-1 are key in regulating immune activation in normal cells as they allow suppression of activated T cells after an initial response to antigen. Tumor cells exploit this mechanism by expressing immunosuppressive markers such as PD-L1. PD-L1 causes T cell anergy by binding to PD-1, resulting in decreased release of IL-2, a cytokine responsible for activating T cells. In ovarian cancer, PD-L1 expression on monocytes in patient ascites and blood samples correlates with poor clinical outcome. Another immune checkpoint, cytotoxic T-lymphocyte-associated protein 4 (CTLA-4), expressed on the surface of T cells binds to the co-stimulatory factor CD80 on antigen-presenting cells, inhibiting T cell activation and causing cell-cycle arrest [29].

Anti-PD-1 and anti-CTLA-4 treatments, termed “immune checkpoint blockade” therapy, were first approved by the FDA for treatment of malignant melanoma in 2011, and for non-small cell carcinoma and renal cell carcinoma in 2014. In ovarian cancer, most trials incorporating immune checkpoint blockade as single agent therapy found an objective response rate of 10–15%. The reason for this poor response could be related to high PD-L1 expression in ovarian cancer, as well as the relatively low amount of tumor-infiltrating cells in the cancer microenvironment [30]. Ovarian cancer has a low mutational burden, potentially causing fewer activated T cells in the tumor milieu [31]. A phase I trial (NCT00729664) with 17 ovarian cancer patients treated with a PD-L1 blocking antibody (BMS-936559) resulted in a partial response in one patient and disease stabilization in two others. In a phase II study (UMIN000005714) using nivolumab (anti-PD-1), 10% of patients with platinum-resistant ovarian cancer demonstrated durable complete response, with a partial response in 15% of those enrolled [32]. Under the KEYNOTE-028 multicohort phase Ib study, the anti-PD-1 agent pembrolizumab was administered to platinum-resistant ovarian cancer patients, achieving an objective response rate of 11.5%, with 23% of the patients exhibiting stable disease [30]. Ipilimumab, which blocks CTLA-4, was used in a phase II study as a single agent for patients with platinum-sensitive ovarian cancer (NCT01611558). Ninety-five percent of the patients did not complete the induction phase due to disease progression, drug toxicity, death, or other unreported causes. Interestingly, patients with hypercalcemic small cell carcinoma, a rare and highly aggressive type of ovarian cancer, have a more active immune microenvironment, express high levels of PD-L1, and respond better to immunotherapy. In a recent clinical trial, after undergoing optimal tumor resection, four patients underwent additional chemotherapy as well as anti-PD1 therapy. Seventy-five percent remained disease free for 1.5 years or more, while the rest experienced a partial response for 6 months [33]. Hypercalcemic small cell carcinoma is driven by mutations in *SMARCA4*, which encodes the BRG1 chromatin regulator. It is as yet unclear whether the changed epigenetic environment of these tumors contributes to the more active immune environment. In conclusion, while checkpoint blockade therapies have shown durable, long-lasting responses in solid tumors like melanoma, lung cancer, and renal cell carcinoma, they have yielded little success in ovarian cancer [33]. Across solid tumors, patients with fewer infiltrating lymphocytes and lower neoantigen burden show very poor response rates to immunotherapy [34]. The immunologically cold nature of most ovarian cancers likely explains why immune checkpoint inhibitors alone provide limited response in ovarian cancer, providing a rationale for combining other targeted therapies with immune checkpoint blockade therapy in this disease.

Other therapies targeting the immune response have shown promise in ovarian cancer. A phase II study (NCT00326885) employed catumaxomab, a tripartite antibody that recruits and activates immune effector cells. This antibody includes paratopes against the epithelial cell adhesion molecule (EpCAM) to target tumor cells, an anti-CD3 paratope enabling binding to T cells, and an Fc-specific component inducing natural killer (NK) cell-mediated cytotoxicity and tumor localization by T cells. Patients on catumaxomab experienced significant benefit from the therapy with an increase in overall survival of 111 days [35]. However, progression-free survival and objective response rate were not assessed in this study, making it difficult to draw conclusions about the use of catumaxomab as first-line treatment. Therapies targeting cytokines also show limitations in treating ovarian cancer. Pro-inflammatory cytokines such as IL-12 have demonstrated promising pre-clinical results in provoking an immune response. However, IL-12 expressing plasmids interperitoneally administered to patients on chemotherapy did not improve response when compared to chemotherapy alone [36]. Upregulation of the intracellular enzyme indoleamine 2,3 dioxygenase (IDO) can diminish the immune response by metabolizing tryptophan into kynurenine, a toxic byproduct that can deplete NK cells and promote T regulatory cells, suppressing an immune response and thus promoting tumor escape [28, 36]. A phase I trial (NCT01191216) combining the IDO inhibitor indocimod with docetaxel in patients with various metastatic solid tumors found that 41% had stable disease, with 18% of patients exhibiting a partial response (PR) compared to the control group [37]. However, a phase II study comparing an IDO1 inhibitor to tamoxifen to target recurrent epithelial ovarian cancer, primary peritoneal carcinoma, and fallopian tube carcinoma was terminated early due to lack of significant difference between responses in treatment and control groups [38].

In ovarian cancer and most other solid tumors, there is a positive correlation between tumor mutational load and the presence of neoantigens on tumors (reviewed in [39]). This concept has recently been exploited to create vaccines that increase the tumor-associated antigen (TAA) presentation on antigen-presenting cells (APCs) to incite a strong tumor-specific immune response. This, in turn, produces tumor antigen-specific cytotoxic T lymphocytes (CTLs) and with it a long-lasting immunologic memory against the tumor. CAN-003, a phase II study on 63 patients with epithelial ovarian cancer, administered the Mucin-1 (MUC1)-derived dendritic cell vaccine Cvac, with notable increases in progression-free survival and objective survival compared to a mock vaccine (NCT01068509). This vaccine targets MUC1, a tumor-associated antigen that is overexpressed in colorectal, pancreatic, and ovarian cancer (reviewed in [39]). The most recent dendritic cell-based vaccine clinical trial in ovarian cancer in the United States ended in 2016, using dendritic cells loaded in vitro with autologous tumor cell lysates (NCT01132014). A similar study conducted in Switzerland on a smaller cohort of patients with recurrent ovarian cancer tested intranodal autologous dendritic cells pulsed with oxidized autologous whole-tumor cell lysate, either as a single agent or in combination with bevacizumab. This study found an amplified T cell response in the vaccine group, yielding significantly prolonged survival [40]. Clinical trials have also tested the administration of tumor antigens as vaccines. Similar to MUC1, the cancer testis antigen NY-ESO-1 is a vaccine target in epithelial ovarian cancer, promoting induction of tumor-specific humoral and cellular responses. In a phase II trial (NCT00616941), the NY-ESO-1 vaccine activated CD8^+^ T cell responses in 32% of patients, with a median PFS of 21 months and a medial overall survival of 48 months [41]. Despite these initial promising results, the heterogeneous nature of ovarian cancer can make it difficult to select the right tumor antigen to administer as a vaccine, suggesting that a multi-targeted approach might be more effective in reversing tumor immune escape [42].

### Epigenetic aberrations in ovarian cancer

The clear limitations of the therapies reviewed above in the treatment of ovarian cancer set the stage for the use of novel epigenetic therapies to treat this disease, either alone or in combination with other therapies. The epigenetic modification DNA methylation regulates gene expression through the addition of a methyl group to promoter regions of DNA to silence gene expression. This is especially crucial during sensitive cellular processes like embryonic development, X-chromosome inactivation, and genomic imprinting, in which a precise balance of active and silenced genes determines the normal development and maturation of an organ [36]. Cancer, on the other hand, promotes global hypermethylation of promoter-associated CpG islands, which are generally unmethylated in normal tissue, silencing genes important for cellular homeostasis such as tumor suppressor genes. Aberrant DNA methylation and structural chromatin changes can alter gene expression to promote tumorigenesis [43]. DNA methylation induces a repressive and tightly knit chromatin structure, which can reduce the expression of genes involved in DNA repair, apoptosis, differentiation, drug resistance, angiogenesis, and metastasis. Hypermethylation of gene promoters in cancer causes downregulation of genes involved in cell-cycle regulation, including *BRCA1*, *CDKN2A*, *RASSF1A*, *LOTI*, *DAPK*, *and ICAM-l*. Accordingly, extensive loss of CpG hypermethylation in ovarian cancer correlates with growth inhibition of cancer cells [44].

Hypermethylation of specific gene promoters has been identified in ovarian cancers. Promoter hypermethylation of tumor suppressors *BRCA1* and *RASSF1A* was significantly higher in ovarian cancers compared to non-neoplastic tissues [45]. Analogous to the mutations in *BRCA1* discussed previously, this hypermethylation silences expression to inhibit BRCA1 function, driving genomic instability in ovarian cancers. *RASSF1A* encodes a protein that regulates the cell cycle; silencing of this gene thus promotes cell-cycle progression and uncontrolled cell growth. Gloss et al. demonstrated that the lincRNA ZNF300P1, a crucial regulator of cell cycle and cell motility, is often silenced in serous ovarian cancer by hypermethylation and this methylation can  reliably distinguish tumors from normal ovarian surface epithelium [46]. This is an example of an epigenetic alteration that has promise as a biomarker for disease. Hypermethylation is also observed in clear cell ovarian cancer; 22 different CpG loci associated with nine genes were hypermethylated in clear cell carcinoma [47]. However, clear cell carcinomas also lack methylation of the gene encoding the transcription factor HNF1B, while this gene is methylated in high-grade serous ovarian cancers [48]. Makarla et al. found that hypermethylation of a panel of eight cancer-related genes (*p16, RARβ, E-cadherin, H-cadherin, APC, GSTP1, MGMT,* and *RASSF1A*) was significantly higher in invasive carcinomas compared to non-invasive cancers and benign cystadenomas [49]. Thus, methylation may promote invasion and metastasis of ovarian cancers.

Beyond DNA methylation, chromatin modifying enzymes are altered in ovarian cancers. High levels of the H3K9 methyltransferase G9a, which adds methyl groups to histones (H3K9) to promote a compact chromatin structure and silence genes, were correlated with late-stage, high-grade, and serous ovarian cancer, as well as shorter survival in ovarian cancer patients [50]. Genes marked by the activating H3K4me3 and the silencing H3K27me3 chromatin modifications are described as “poised” or bivalent; these are not transcribed in embryonic stem cells but resolve into active and transcribed (H3K4me3) or silenced and not transcribed (H3K27me3) as stem cells differentiate. These bivalent chromatin loci were silenced in 499 high-grade serous ovarian cancers compared to eight normal fallopian tube samples and included genes in the PI3K and TGF-beta signaling pathways. Ovarian cancer stem-like cells and chemo-resistant ovarian cancer cells showed increased silencing of these genes [51]. As previously described, the gene encoding the ARID1A chromatin remodeler is mutated in over 50% of ovarian clear cell carcinomas. Bitler et al. showed that inhibiting the EZH2 methyltransferase, which adds the H3K27me3 mark to silence gene expression, caused regression of ARID1A-mutated tumors in a mouse model of ovarian cancer. This occurred through upregulation of PIK3IPI, a target of ARID1A and EZH2 that is increased by EZH2 inhibition and inhibits oncogenic PI3K/Akt signaling [52].

Another group of epigenetic modulators targeted for cancer therapy are histone deacetylase (HDAC) enzymes, which remove acetyl groups from histone and non-histone proteins, downregulating the transcription of genes. Deacetylation of positively charged histones causes them to remain tightly bound to the negatively charged DNA, promoting a closed chromatin structure and preventing gene transcription [53]. HDACs are divided into 4 classes: class I, located in the nucleus, contains HDAC1/2/3/8; class II, comprised of HDAC4/5/6/7/9/10, located in both the nucleus and cytoplasm; and class IV, containing HDAC11. Class III includes sirtuins (SIRT 1-7) and, unlike the other HDAC classes, utilizes NAD^+^ rather than Zn^2+^ and thus is not affected by HDAC inhibitors (HDACis) that bind to a zinc moiety. Class III HDAC enzymes are structurally similar to the Sir2 yeast protein, which plays an important role in genome stability [54]. In cancers, HDAC inhibitors can reverse transcriptional repression of tumor suppressor genes and promote an anti-tumor environment.

Specific HDAC inhibitors may show efficacy in tumors with mutations in epigenetic regulators. Bitler et al. demonstrated that ARID1A, a subunit for the SWI/SNF complex which is mutated in more than 50% of ovarian clear cell carcinomas, transcriptionally represses HDAC6. By inhibiting HDAC6 using small molecule ACY1215, they found significant improvement in the survival of mice with ARID1A mutated tumors as HDAC6 deacetylates Lys120 on P53, thus inactivating the proapoptotic function of P53 [55]. Inhibition of HDAC6 in ARID1A-mutated tumors promotes acetylation of P53, restoring its apoptotic and tumor suppressor functions. In addition to specific HDAC inhibitors, pan-HDAC inhibitors have shown significant activity against ARID1A-mutated tumors in preclinical models of clear cell carcinoma [56].

Epigenetic therapies can trigger an immune response in solid tumor cell lines and mouse models [57, 58]. In particular, DNMTis and HDACis can reverse immune evasion and sensitize to subsequent immune checkpoint blockade by inducing an interferon response via upregulation of surface tumor antigens and key immunomodulatory proteins such as major histocompatibility complex (MHC) molecules [36]. In a murine ovarian cancer model, the combination of the DNMT1 inhibitor 5-azacytidine (AZA) and the class I HDACi ITF2357 significantly increased the number of T cells recruited to the tumor by upregulating the expression of endogenous retroviruses (ERVs), ancient viral DNA that composes 8% of our genome, activating a type I interferon response [59]. In ovarian cancer cell lines, AZA removed methylation from ERVs, allowing for their expression. Subsequently, sensing of ERV dsRNA caused activation of that recognize pathogenic molecules such as viral dsRNA. This in turn led to expression of cytokines and activation of an innate immune response. This type I interferon response was further potentiated when AZA was combined with class I and class II HDACis as well as anti-PD-1 immune checkpoint blockade, causing an increase in T cells and natural killer cells in the tumor microenvironment. This correlated with an increase in overall survival in the murine ovarian cancer model [59]. These observations bear clinical significance when trying to combat the immunologically cold nature of ovarian cancer, particularly since the presence of intratumoral T cells may accurately predict both response to immune checkpoint blockade therapy and progression-free survival [60–62].

### Clinical trials of epigenetic modifiers in ovarian cancer

The use of DNMTis and HDACis as cancer therapeutics began in the early 2000s, mainly as treatment for hematological disorders. 5-Azacytidine (AZA) and 5-aza-2′-deoxycitidine (decitabine) were approved by the FDA in 2004 and 2006 for myelodysplastic syndrome (MDS), while the HDAC inhibitor suberoylanilide hydroxamic acid (SAHA) was approved in 2006 for the treatment of persistent or cutaneous T cell lymphoma [43]. Initially, epigenetic treatment was given in a clinical trial setting either alone or in combination with the standard of care to either resensitize the tumor to anticancer therapy or to prevent the development of resistance to therapy. Eventually, these drugs were tested against solid tumors, including clinical trials of both SAHA and AZA in platinum-resistant ovarian cancer. While no antitumor activity was found after treatment with SAHA, AZA demonstrated partial response, but was associated with severe adverse effects including fatigue and myelosuppression [63]. In a different study, the HDAC inhibitor belinostat was administered to platinum-resistant ovarian cancers, but similarly caused severe adverse events such as neutropenia, thrombocytopenia, and vomiting, leading to the termination of the study with no therapeutic benefit over standard treatment [43]. Similarly, in a phase I study, the pan-HDACi vorinostat was administered with carboplatin or gemcitabine and caused severe hematological toxicities despite the partial response observed, leading to the study’s termination [64].

DNMTis have had success in the setting of chemo-resistant ovarian cancer, restoring platinum sensitivity in patients refractory to standard chemotherapy. The DNMTi decitabine sensitizes ovarian cancer patients to platinum-based therapy. DNA hypomethylation caused by decitabine correlates with clinical outcome and is associated with good prognosis. Work by Nephew, Matei, and colleagues has shown that platinum resistance is heavily driven by epigenetic aberrations including abnormal DNA methylation. These abnormal epigenetic changes were successfully reversed in a clinical trial by decitabine administered for 5 days, which was then followed by carboplatin [65]. Demethylation and re-expression of genes in pathways including DNA repair and immune activation may be responsible for resensitization to carboplatin treatment [66]. Decitabine proved more beneficial than 5-azacytidine for platinum-resistant ovarian cancer patients, producing a 35% objective response rate and a 10.2 month PFS that correlated with the degree of demethylation of the tumor suppressor genes *MLH1, RASSF1A, HOXA10, and HOXA11,* [65]. Decitabine has also been shown to alter the methylation status of the tumor antigen NY-ESO-1, potentially allowing for increased expression of this known target for immunotherapy. In addition, decitabine affects pathways known to promote tumor progression such as the sonic hedgehog (Hh) and TGF-β signaling pathway. Hypomethylation caused by decitabine increases the expression of Hh antagonists and alters TGF-β expression, both resulting in improved response to platinum therapy [47].

Thus, while ovarian cancers exhibit changes in epigenetic modifiers and epigenetic regulation of many genes, clinical trials with single-agent epigenetic therapy have been disappointing in this disease. Pre-clinical work has focused on the combination of several epigenetic drugs. The pan-HDAC inhibitor vorinostat was not beneficial in patients with ovarian cancer, with only 1 out of 27 patients achieving partial response. However, when used pre-clinically in combination with the pan-HDAC inhibitor belinostat, the DNMTi decitabine elicited greater platinum re-sensitization of ovarian cancers in a xenograft model when compared to decitabine alone [36]. The rationale for combining DNMTis and HDACis lies in their mechanistic synergy, as opening of chromatin and re-expression of aberrantly silenced genes by DNMTis is further augmented by the action of HDACis, mainly due to inhibition of nuclear HDAC1 and HDAC2. Combination therapy also allows for a reduction of the toxicity profile of these drugs by significantly reducing the dose administered to yield comparable effects. However, a study conducted in 2013 by Falchook et al. using 5-azacytidine, valproic acid (a pan-HDACi), and carboplatin in platinum-resistant epithelial ovarian cancer demonstrated that this combination is highly toxic, with 80% of participants experiencing grade 3 or higher adverse effects including fatigue, vomiting, and neutropenia, thus forcing the study to terminate early [67]. It is important to note that the toxicity profiles of valproic acid and 5-azacytidine are quite severe as their targets span the epigenome, stressing the need for more selective epigenetic modulators. Despite the rather discouraging results of this study, combination therapy remains an attractive therapeutic goal, provided selective agents with non-synergistic toxic profiles are combined. In another solid tumor, non-small cell lung cancer, combining DNMTis and HDACis showed durable and robust clinical responses in several patients [36].

Combining immunotherapy with DNMTis and/or HDACis allows for the activated immune system to act unchecked against the tumor cells. Preclinical studies support the rationale for combining epigenetic and immune therapies: in a syngeneic ovarian cancer mouse model, a combination of decitabine and anti-CTLA-4 significantly reduced tumor growth and prolonged survival compared to each drug alone [68]. Decitabine increased the expression of chemokines, recruiting natural killer cells and CD8 cells to the tumor microenvironment. This prolonged the cytotoxic lymphocyte response, which increased mouse survival. These preclinical results have led to trials combining epigenetic therapies with immune checkpoint blockade in ovarian cancer (Additional file 1). In non-small cell lung cancer, patients on DNMTi coupled with a class 1 HDACi, who progressed despite epigenetic therapy, mounted a strong and durable response after treatment with immune checkpoint therapy, encouraging the use of triple combination therapy in solid tumors [69]. Strides are being made towards combination therapy in ovarian cancer in both preclinical settings and clinical trials. Currently, a phase Ib/II clinical trial in patients with chemo-resistant epithelial ovarian cancer is testing the combination of entinostat, a class 1 HDACi (HDAC1/3 inhibitor), together with avelumab, a humanized antibody targeting PD-L1, showing initial promising results (NCT02915523). Odunsi et al. found that expression of the cancer testis antigen NY-ESO-1 is silenced by DNA methylation. When they paired a vaccine against this antigen with decitabine and doxorubicin, disease stabilization and partial clinical response occurred in 6 out of 10 patients with relapsed epithelial ovarian cancer [41].

In addition to DNMTs and HDACs, epigenetic modulators being targeted in clinical trials include the histone lysine methyltransferase EZH2 and BET proteins, which are composed of BRD2, BRD3, BRD4, and BRDT and contain bromodomains that recognize acetylated lysine residues on histone tails [70, 71]. While EZH2 inhibition for ovarian cancer is currently in preclinical stages, various clinical trials are ongoing for other solid tumors including lymphomas (NCT01897571). BET inhibition suppresses MYC, an oncogene whose expression is positively regulated by BRD4. In ovarian cancer, BRD4 is frequently overexpressed and correlates with poor prognosis. Inhibiting BET induces cell-cycle arrest and inhibition of tumor formation [70]. When combined with PARP inhibitors, BET inhibitors enhance PARP inhibitor-induced DNA damage in cancer cells, most likely by reducing homologous recombination, a DNA damage repair mechanism [72]. BET inhibitors are also being evaluated with immune checkpoint blockade: a phase Ib open label trial is currently testing BET inhibitor RO6870810 and atezolizumab (anti-PD-L1) in patients with advanced ovarian cancer or triple negative breast cancer, to be completed in July 2020 (NCT0329217). Additional file 1 lists completed and ongoing clinical trials utilizing epigenetic modifiers in ovarian cancer, either alone or in combination with other therapies including PARP inhibitors and immunotherapies.

## Conclusions

**Fig. 1:**
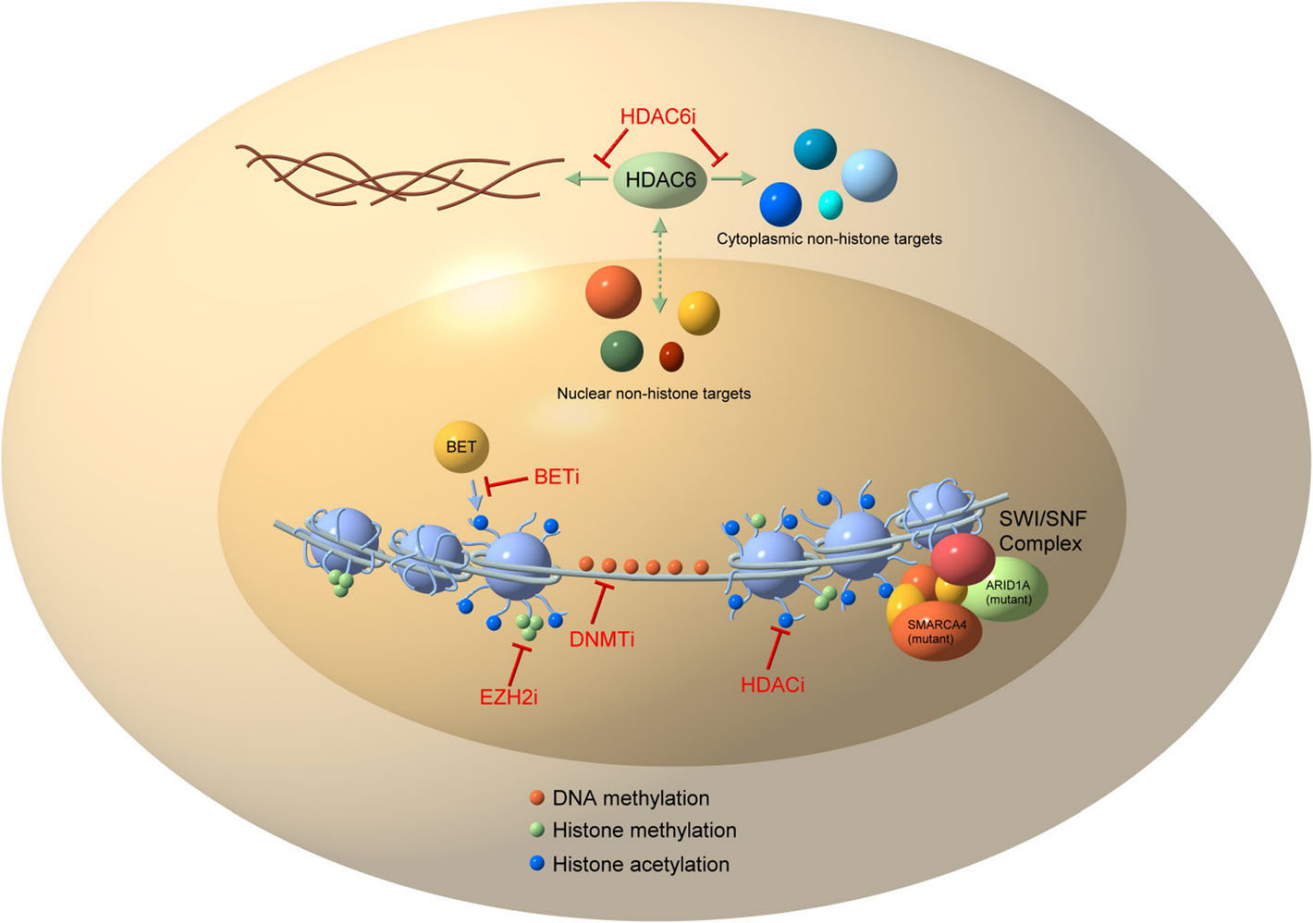
Targeting epigenetic modifiers in ovarian cancer. DNA (light blue line) is wrapped around histones (light blue spheres). Modifications to histones (methylation, acetylation) and DNA (methylation) affect gene expression. The SWI/SNF complex (components of which are commonly mutated in ovarian cancers, especially ARID1A and SMARCA4) remodels chromatin and mutations in these proteins change the epigenetic landscape. Drugs targeting BET proteins (BETi), histone methylation (EZH2i), histone acetylation (HDACi), and DNA methylation (DNMTi) change gene expression in cancers and are currently being explored as therapeutic options for ovarian cancer in clinical trials. Separately, inhibition of HDAC6 affects non-histone targets

Ovarian cancer tumorigenesis is heavily mediated by epigenetic changes (Fig. 1), and disease progression, response to therapy, and immune tolerance are directly affected by aberrant DNA methylation and histone modifications. Modern cancer therapies are being tailored to fit these recent discoveries. The administration of intraperitoneal chemotherapy has been the only modification to traditional ovarian cancer treatment shown to improve overall survival; however, this does not eliminate toxicities associated with traditional chemotherapy [73]. Due to low response rates to single-agent epigenetic, immune, and targeted therapy, improved combination treatments are urgently needed in ovarian cancers  and are the focus of several exciting new clinical trials.

We envision that the future of effective therapy for ovarian cancer will have to rely on (1) a combination of several therapies based on strong pre-clinical results and (2) a targeted approach based on the mutational background of the tumor (for example, HDAC6 inhibitor treatment of ARID1A mutated tumors). As discussed above, ovarian cancer has a complex histological and molecular expression pattern, leading to inconsistent results when using a single class of drugs. Understanding the molecular makeup of each tumor will allow for the appropriate use of epigenetic drugs, immunomodulators, targeted therapies, and combinations of these to prevent tumor growth. Thus, more emphasis should be placed on biobanking and analyzing patient tumor samples prior to initiating a treatment regimen. This will allow for more effective use of combination therapies, applied based on both the molecular subtype and the tumor immune microenvironment. The results of ongoing clinical trials will shed light on combined pharmacodynamics and multifactorial mechanisms of epigenetic drugs when used together or in combination with targeted (for example, PARP inhibitors) or immune (for example, anti-PD-1) therapies to significantly improve treatment for this deadly disease.

## Additional file

Additional file 1: Completed and ongoing clinical trials utilizing epigenetic modifiers in ovarian cancer. (XLSX 17 kb)

## Abbreviations

APC: Antigen-presenting cell
AZA: 5-azacytidine
BRCA1/2: Breast Cancer Susceptibility Gene 1/2
CTL: Cytotoxic T lymphocyte
CTLA-4: Cytotoxic Tlymphocyteassociated Protein 4
DNA: Deoxyribonucleic acid
DNMT: DNA methyltransferase
DNMTi: DNMT inhibitor
EGFR: Epidermal growth factor receptor
EOC: Epithelial ovarian cancer
ERV: Endogenous retrovirus
FDA: Food and Drug Administration
HDAC: Histone deacetylase
HDACi: HDAC inhibitor
HGSC: High-grade serous (ovarian) carcinoma
Hh: Sonic hedgehog (signaling pathway)
IDO: Indoleamine 2,3 dioxygenase
IL-12: Interleukin-12
LGSC: Low-grade serous (ovarian) carcinoma
MAPK: Mitogen-Activated Protein Kinase
MMR: Mismatch repair
mOC: Mucinous ovarian carcinoma
MUC1: Mucin-1 protein
NAD: Nicotinamide adenine dinucleotide
NCCN: National Comprehensive Cancer Network
NK: Natural killer (cell)
PARP: Poly(ADP-ribose) polymerase
PD-1: Programmed cell death protein 1
PD-L1: Programmed cell death ligand1
PFS: Progression-free survival
PR: Partial response
RNA: Ribonucleic acid
SAHA: Suberoylanilide hydroxamic acid
TAA: Tumor-associated antigen
TCGA: The Cancer Genome Atlas
TGF-ß: Transforming growth factor beta
TLR: Toll-like receptor
VEGF: Vascular endothelial growth factor
VEGF-A: A member of the VEGF growth factor family
VEGF-R: VEGF receptor
